# Metal-Organic Framework-Based Engineered Materials—Fundamentals and Applications

**DOI:** 10.3390/molecules25071598

**Published:** 2020-03-31

**Authors:** Tahir Rasheed, Komal Rizwan, Muhammad Bilal, Hafiz M. N. Iqbal

**Affiliations:** 1 School of Chemistry & Chemical Engineering, Shanghai Jiao Tong University, Shanghai 200240, China; masil@sjtu.edu.cn; 2 Department of Chemistry, University of Sahiwal, Sahiwal 57000, Pakistan; Komal.rizwan45@yahoo.com; 3 School of Life Science and Food Engineering, Huaiyin Institute of Technology, Huaian 223003, China; 4 School of Engineering and Sciences, Tecnologico de Monterrey, Campus Monterrey, Ave. Eugenio Garza Sada 2501, Monterrey, NL CP 64849, Mexico

**Keywords:** metal-organic frameworks, porous materials, reaction coordination, biomedical applications

## Abstract

Metal-organic frameworks (MOFs) are a fascinating class of porous crystalline materials constructed by organic ligands and inorganic connectors. Owing to their noteworthy catalytic chemistry, and matching or compatible coordination with numerous materials, MOFs offer potential applications in diverse fields such as catalysis, proton conduction, gas storage, drug delivery, sensing, separation and other related biotechnological and biomedical applications. Moreover, their designable structural topologies, high surface area, ultrahigh porosity, and tunable functionalities all make them excellent materials of interests for nanoscale applications. Herein, an effort has been to summarize the current advancement of MOF-based materials (i.e., pristine MOFs, MOF derivatives, or MOF composites) for electrocatalysis, photocatalysis, and biocatalysis. In the first part, we discussed the electrocatalytic behavior of various MOFs, such as oxidation and reduction candidates for different types of chemical reactions. The second section emphasizes on the photocatalytic performance of various MOFs as potential candidates for light-driven reactions, including photocatalytic degradation of various contaminants, CO_2_ reduction, and water splitting. Applications of MOFs-based porous materials in the biomedical sector, such as drug delivery, sensing and biosensing, antibacterial agents, and biomimetic systems for various biological species is discussed in the third part. Finally, the concluding points, challenges, and future prospects regarding MOFs or MOF-based materials for catalytic applications are also highlighted.

## 1. Introduction

**Figure 1:**
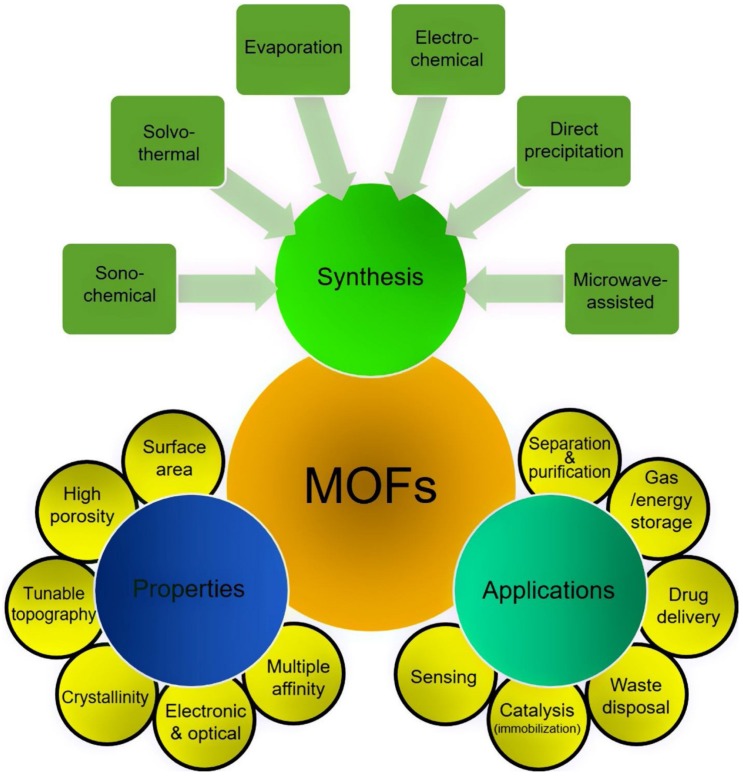
A schematic overview of MOF synthesis, properties, and applications. Reprinted from Bilal et al. [14] an open access article under the CC BY-NC-ND license (http://creativecommons.org/licenses/by-nc-nd/4.0/). Copyright (2018) Brazilian Metallurgical, Materials and Mining Association. Published by Elsevier Editora Ltda.

From the last several years, metal-organic frameworks (MOFs) with their unique catalytic functionalities have emerged as an attractive class of crystalline materials on the interface of material science and coordination chemistry [1,2]. They are the self-assembled organic-inorganic hybrid units having polynuclear secondary building units (SBUs) or metal nodes, which form a porous and periodic framework. These units have the coordination preference between metal ions, rigidity, and length of the organic ligands [3]. Their unique structural topologies, extraordinary surface area, ultrahigh porosity, and diverse range of applications make the materials promising for applied research, addressing issues found in catalysis, proton conduction, gas storage, drug delivery, sensing, and separation [4,5,6,7]. Currently researchers are using several functionalization methodologies for MOFs, such as post-synthetic modification. These modifications can be carried out by constructing the metal clusters or organic ligands with the help of functional ligands, embedding some useful molecules, such as organometallics, metal nanoparticles, enzymes, heteropoly acids, etc. within the pores [8,9]. The phenomenal properties can be governed to the MOFs materials by various species, including inorganic metal ions, organic ligands, and guests. These multi-functional sites further enhance interest in MOFs materials. Presently, four well-established methodologies are being used to develop functional sites in the MOF architecture such as organic ligands, the introduction of various guest components, both metal clusters and organic ligands, and constructing an array with functional sites which include guest molecules with another functional site [10,11,12,13,14]. These approaches make MOFs an optimum platform to design and manufacture functionally fabricated materials. Figure 1 illustrates a schematic representation of various synthesis routes, considerable properties, and applications of MOFs [14].

From the catalysis perspectives, MOFs materials present fascinating advantages, as catalysts, over others in practice, and conventional catalytic materials, such as clays, zeolites, or mesoporous silica. Among several notable multifunctional catalytic potentialities of MOFs, some can be highlighted: (1) the structural tunability, (2) diversity of MOF components, i.e., linkers, nodes, and pores, (3) various catalytic sites in a single MOF, (4) the highly tunable and uniform porous environments, (5) recognition and transport of products and substrates, (6) the well-defined and adjustable framework allows to comprehend the catalytic mechanism, (7) structure-function relationship at the molecular level, and (8) rigorous catalysis in a wide array of catalytic reactions [6,13,14].

Considering the abovementioned points, the present work emphasizes the noteworthy multi-catalytic potentials and recent applications of MOFs, with particular reference to catalysis chemistry perspectives. A specific focus is given to electrocatalysis, photocatalysis, and biocatalysis features of MOFs with suitable examples in different sections. In the first part, we discussed the electrocatalytic behavior of various MOFs, such as oxidation and reduction candidates for different types of chemical reactions. The second section of the review focuses on the photocatalytic behavior of various MOFs as potential candidates for light-driven reactions, including photocatalytic degradation of various contaminants, CO_2_ reduction, and water splitting. The third part of the review discusses the use of MOFs based porous materials in biomedical applications such as drug delivery, sensing and biosensing, antibacterial agents, and biomimetic systems for various biological species. Lastly, the conclusions, challenges, and future prospects regarding MOFs or MOF-based materials for catalytic applications are also highlighted.

## 2. Structural Classification and Synthesis of MOFs

MOF development depends upon their synthesis and post-synthesis modifications, but they are primarily classified as normal MOFs (1^st^ generation), functional MOFs (2^nd^ generation), and smart MOFs (3^rd^ generation). First-generation MOFs were designed based on molecular architecture organic and inorganic moieties. The 2^nd^ generation MOFs were derived from 1^st^ generation MOFs by some chemical modifications. Similarly, 3^rd^ generation MOFs were constructed by conjugation of biomolecules like some organic drugs, cations, toxins, gases, and certain bioactive molecules [15]. MOFs are also categorized based on the nature of the chemical structure of the framework, either rigid or flexible. Because the structural orientation changes against external strain such as molecular enclosure, or limiting factors (pH, temperature) of some kinds of MOFs are irreversible, these kinds of MOFs are referred to as rigid MOFs. In contrast to rigid MOFs, the structural conformation of the flexible MOFs can be changed in response to external stimuli [4]. In MOFs, crystal structure plays an imperative role in functional integration. Therefore, MOFs can also be classified into amorphous and crystalline based on their crystal structure. In chemistry, crystal structures have infinite arrangements of high confined structures. Similarly, crystalline MOFs show the same properties as formal crystal structure. On the other hand, amorphous MOFs consist of basic building moieties similar to the crystalline structure, but they have different long-range periodic structural conformation arrangements [16].

**Figure 2:**
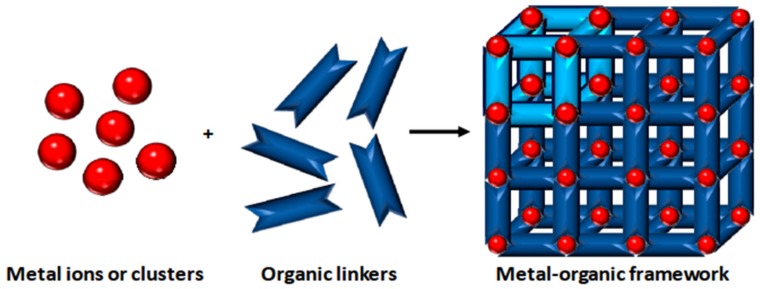
Scheme for the preparation of a MOF. Different metal ions or clusters are mixed with organic linkers using a suitable solvent. Coordination polymerization takes place between the precursors, resulting in a cross-linked network showing potential voids. Reprinted from Carrasco [17] an open-access article distributed under the terms and conditions of the Creative Commons Attribution (CC BY) license (http://creativecommons.org/licenses/by/4.0/). Copyright (2018), the author. Licensee MDPI, Basel, Switzerland.

In the synthesis of MOFs, organic linkers (fumaric acid, propanedioic acid, ethanedioic acid, and benzene-1,3-tricarboxylic acids) connect the metal building blocks through coordination bonds (Figure 2) [17]. The strength of a bond is defined as the symmetrical shape, confined geometrical arrangement, and high crystalline structure, which play a vital role in establishing the ultimate physiochemical properties of structure [18]. Despite the use of traditional porous materials in the synthesis of MOF, the porous structure of MOFs can be tweaked by geometrical fine-tuning between metal ions and organic linkers. In some cases, various chemically active molecules are attached during the activation process either at the peripheral or core of MOFs in the PSM stage that can alter the functional behavior (absorption, increase specificity) [19]. There are several methods for the synthesis of MOFs, for example, electrochemical synthesis, solvothermal methods, microwave synthesis, mechano-chemical synthesis, and sonochemical synthesis [20]. PSM has been used to alter the function of MOFs by adding a functional group. These functional groups could be attached with a covalent bond (zirconium-based MOFs) and a non-covalent bond depending upon the application [21,22].

## 3. Metal-Organic Frameworks (MOFs) as Active Cues in Electrocatalysis

During recent decades, electrochemical devices, including fuel cells, batteries, capacitors, and electrolytic cells, have appealed much attention [23,24,25,26]. A variety of half-cell reactions, for example, (1) hydrogen evolution reaction (HER), (2) hydrogen oxidation reaction (HOR), (3) oxygen evolution reaction (OER), (4) oxygen reduction reaction (ORR), (5) CO_2_ reduction (CO_2_RR), (6) sulfur redox, (7) metal oxidation and (8) metal ion reduction take place in electrochemical devices. Surplus energy and large over-potential require for carrying out few half-cell reactions (HER, HOR, OER, ORR, CO_2_RR). Such reactions usually possess slow reaction kinetics and provide lower energies and reaction potentials than the theoretical calculations. Platinum (Pt) plays a significant role in adsorption and dissociation phenomena of H_2_ in the half-cell and the reverse reactions of HER and HOR [27]. Difficulty in reaction selectivity in few cell reactions (ORR and CO_2_RR) provides undesirable products and insufficient utilization of energy. Highly efficient selective catalysts are required to overcome such problems. Unique structural features of MOF as uniform pore sizes/shapes, large porosities, crystalline nature, ordered assortments, and ligands linked by coordination bonds [28,29]. Moreover, the available excess of active sites and redox potential made them valuable for selectivity, catalysis, storage, sensing, drug delivery, and different other purposes [4,30,31,32,33]. This is an urgent need of time to develop unique, clean, sustainable technologies for energy conversion and storage. Based on their unique features, MOF-based materials are being developed as novel electrocatalysts for carrying out oxygen, hydrogen evolution reactions, oxygen and carbon dioxide reductions reactions, and some other different redox reactions. By taking into account the benefits of inorganic/organic homogeneous and heterogeneous catalysts, MOFs can provide an encouraging platform to achieve high electro-catalytic potential, so more attention should be paid to design novel MOFs having high electrical conductivity, chemical stability. MOFs being heterogeneous catalysts possess many kinds of reactive catalytic sites with coordination structures and surrounding environments, which have similarities to proteins and molecular metal complexes [34,35]. The metals and organic ligands present in the MOF structure may play different characters as a base, acid, redox mediator, and catalyzing different reactions [4,36].

### 3.1. Characteristic Features of MOFs as Electrocatalysts

Noble metals, their oxides, and electrocatalysts, which are based on earth-abundant metals, have been examined previously. However, the catalytic potential, abundance, durability, and cost of the noble metal oxides are still the main challenges after decades [37,38]. For improving the catalytic behavior of inorganic catalysts, different strategies, including an increase in surface area, by exposing the more active crystal surfaces, optimization of particle size, and morphology, were employed. During electrochemical processes, heterogeneous catalysts undergo structural evolution [39,40,41]. Soluble metal complexes having particular structures are useful for mechanistic studies with the help of different electrochemical measurements [42]. Thereof, at low concentration, by using all its active catalytic sites, MOF-based homogeneous catalysts provide high activity.

The inner pores of MOFs provide a large surface area, and different crystallographic techniques can be used to visualize the ordered array of active catalytic centers. The environment may cause a change in the structure of insoluble solids, especially MOFs [43,44,45]. Electrocatalytic reactions take place in electrolyte solutions and high acidity or basicity required, so poor chemical stability of MOFs is the main problem in electrolysis phenomena. MOFs having excellent chemical stabilities in H_2_O and acidic/basic media have been developed, and few of them have been successfully employed as electrocatalysts [46,47]. The purpose of designing highly stable MOFs is to increase the bonding strength of metal-ligand coordination, and this can be done either by combining carboxylate ligands with high oxidation state metal ions or by combining very basic azolate ligands with soft metal ions [48]. A decrease in the catalytic potential of MOFs may occur due to hydrophobic materials coating outside the crystals to prevent the nucleophilic attacks of electrolyte species. For electrocatalyst, high electrical conductivity in important phenomena [49], and MOFs are usually poorly electric conducting materials [50,51]. However, this poor conductivity potential of MOFs can easily be overcome by matching the compatibility and surface functionalization.

### 3.2. Oxidation Reaction (Hydrogen Evolution Reaction, HER)

As compared to other fuels, hydrogen is considered as a promising fuel because water is its only combustion product. For producing hydrogen, electrolysis of water is an efficient and eco-friendly method. Pt and Pt-group containing metals are considered as the most efficient electrocatalysts for hydrogen evolution reactions (HER). In 0.5 M H_2_SO_4_ (pH = 0), 20 wt% Pt/C is used as standard (η_10_ = 30 mV). Molybdenum (Mo)-, tungsten (W)-, cobalt (Co)-, and nickel (Ni)-based materials show relatively high potentials in the case of heterogeneous inorganic catalysts [23]. Few homogeneous catalysts as molybdenum (Mo), iron (Fe), cobalt (Co), and nickel (Ni)-based complexes exhibit high turnover frequency (TOF) for hydrogen evolution reactions (HER), this is particularly true for Mo complexes [42,52]. Still, these catalyst complexes suffer from large η and less stability [53,54]. Hydrogen evolution reactions are carried in strong acidic electrolytes. Before the adsorption of hydrogen atom intermediate on the catalyst under neutral/alkaline conditions, it is important for HER to break down the oxygen-hydrogen (O–H) bond [55]. Moreover, in neutral electrolytes, the cell resistance increases, and electrolytic potential get lowers, this may be due to slow movement of buffer ions and pH gradient formation [56].

**Figure 3:**
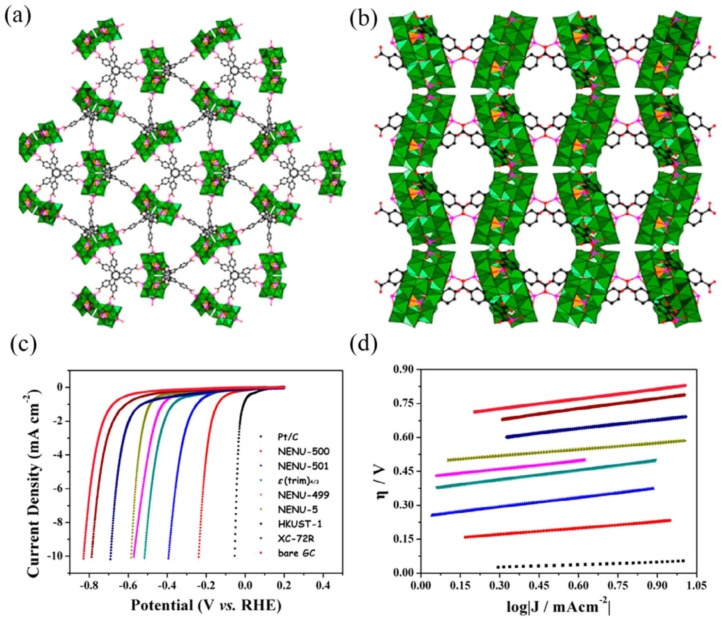
Schematic presentation of (**a**) NENU-500 (**b**) NENU-501 (**c**) LSV curves (**d**) Tafel plots of corresponding electrodes and other related materials in 0.5 M H_2_SO_4_. Reprinted from Qin et al. [58] with permission from the American Chemical Society. Copyright (2015), the American Chemical Society.

Polyoxometalate (POM)-based MOFs with catalytic HER potential (pH = 1) have been reported. Zn-modified ε-Keggin type POMs were used to construct different MOFs [57]. Two novel POM-based MOFs with 3D porous frameworks were synthesized by using Zn-modified ε-Keggin POMs (Figure 3) [58]. In strong acidic media, they showed excellent chemical stability and potent HER activities [58]. POMs were found in isolated and dimerized forms in the NENU-500, NENU-501, respectively. Dichalcogenides as molybdenum, cobalt, and nickel sulfides exhibit potential HER activity as compared with their bulk forms and Pt/C [59]. Those 2D MOFs having similarities with dichalcogenide were also found active as HER catalysts [58,60,61]. MOS-1 and MOS-2 are MOFs, which are honeycomb-like 2D sheets. MOS-1 shows electron conductivity (10^−6^ S cm^−1^), and they are isostructural with [Pt^IV^_3_ (tht)_2_] [62], while MOS-2 possesses larger pores and is isoreticular with MOS-1. HER activity of 2D MOF H_3_[Ni^III^_3_(tht)_2_] nanosheets, which are found isostructural with [Pt^IV^_3_(tht)_2_] has been previously reported [61]. By using the Langmuir-Blodgett strategy, nanosheets having a thickness of 0.7 nm approximately were prepared. Onto the GC-RDE, the samples of nanosheets were horizontally transferred to function as an electrode. HER potentials were assessed at 1600 rpm in 0.5 M H_2_SO_4_ nitrogen saturated solution. The electrode H_3_[Ni^III^_3_(tht)_2_] exhibited a η_10_ of 333 mV and a TS of 80.5 mV dec^−1^ [61].

**Figure 4:**
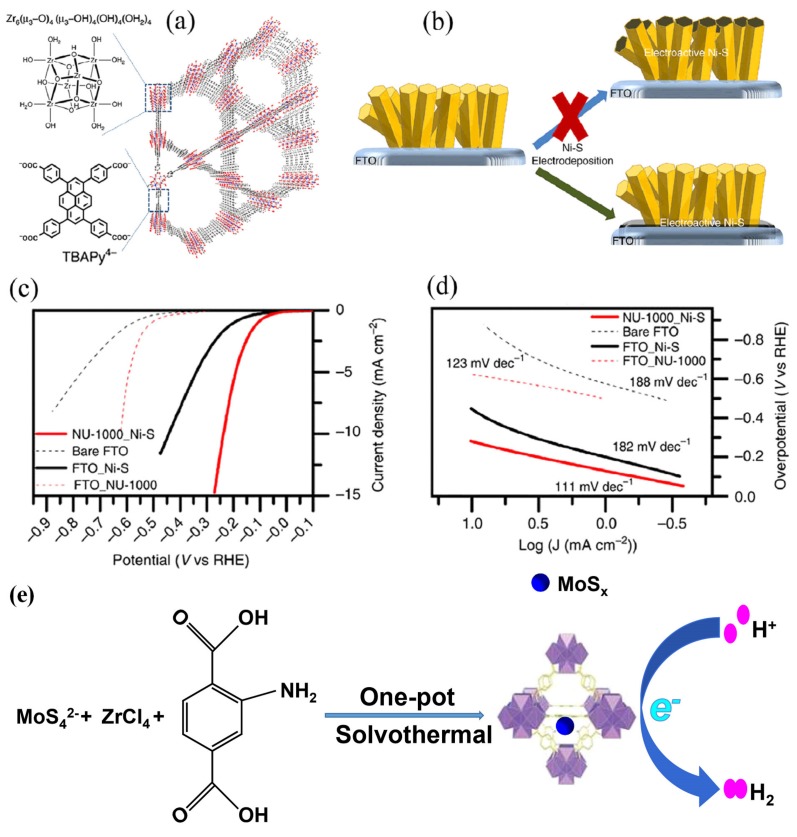
(**a**) Schematic illustration of NU-1000. (**b**) Ni–S Electrodeposition for the formation of the NU-1000/Ni–S hybrid (**c**) LSV curves (**d**) Tafel plots of NU-1000/Ni–S composite and related materials. Reprinted from Hod et al. [64] an open-access article licensed under a Creative Commons Attribution 4.0 International License http://creativecommons.org/licenses/by/4.0/, and (**e**) Presentation of the synthesis of the Zr-MOF stabilized MoSx. Reprinted from Dai et al. [66] with permission from the American Chemical Society. Copyright (2016) the American Chemical Society.

Synthesis and HER potential of 2-D MOF containing CoS_2_N_2_ coordination spheres and mixed ligands have been reported [63]. This MOF was also found isostructural with [Pt^IV^_3_(tht)_2_]. The molar ratio of Co/N/S (2.5:4.8:5.2) was found constant with MOF chemical formula. The excellent solubility of H_6_tha in water (H_2_O) made the synthesis of H_3_[Co^III^_3_(tha)_2_] (THA-Co) unsuccessful, and this renders it’s HER potential incomparable. For HER evaluations in 0.5 M H_2_SO_4_ solution saturated with nitrogen, the synthesized samples of nanosheets were transferred onto the GC-RDE. Electrode THAT-Co showed low η_10_ value of 283 mV, which was lower as compared to THTA-Ni (315 mV) and THT-Co (328 mV), thus the catalytic activity ranking was found to be CoS_2_N_2_ > NiS_2_N_2_ > CoS_4_. Furthermore, a composite of microcrystalline THAT-Co and grapheme was synthesized, which exhibited potently, HER activity with η_10_ value 230 mV than nanosheets THATA-Co electrode with η_10_ value 283 mV. To achieve the synergistic effect for HER activity, MOFs can also play a vital role in holding inorganic catalysts as supporting materials. Hod et al. reported that composite material made of metal sulfide and MOF could play a role of electrocatalyst for HER exhibiting good activity and durability [64]. Ni-S has been reported as an efficient catalyst for HER potential [65]. Dai et al. [66] demonstrated the use of a porous framework where a Zr-based MOF (UiO-66-NH_2_) was combined with molybdenum polysulfide (MoS_x_) to provide HER catalysts. In addition, this catalyst offers high durability and excellent stability (Figure 4) [64,66]. This excellent performance of catalysts may be attributed to the wide catalytic surface area, rich active sites and rapid electron movement from nanosheets of molybdenum polysulfide to electrode.

### 3.3. Reduction Reaction (Oxygen Evolution Reaction, OER)

The oxygen evolution reaction (OER) is an important half-reaction of H_2_O electrolysis, which takes place at the anode. It is complex and slow as compared to HER [67]. Metal oxides and hydroxides are formed in OER, which are not stable in strongly acidic media, except for IrO_2_ and RuO_2_ [68]. Therefore, alkaline conditions as KOH solutions are opted to carry out OER experiments. Some studies reported the OER experiments in neutral media as well. Precious metals-based complexes have fascinated scientists as OER catalysts due to their excellent potential. Various scientists have reported the precious metals based MOFS as potent OER catalysts [69,70].

Porphyrin moieties containing MOFs are highly reactive and have attracted considerable attention as electrocatalyst [71]. For example, Zr(IV) based MOF also called (PCN-224-Ni) comprised of Zr-oxo nodes and Ni(II) porphyrin (Ni(II)TCPP) units was synthesized, which showed OER potential in 0.1 M aqueous solution of NaClO_4_ [72]. After OER activity, PCN-224-Ni structure was reserved. In this, porphyrin-based MOF, Ni(II)TCPP was found responsible for the intrinsic activity and Zr nodes play the role of proton abstractor. This phenomenon confirmed the intrinsic activity of porphyrin moiety. Meso-substituents on porphyrin units may coordinate with second metals to produce heterobimetallic pyridyl-porphyrin MOFs. As compared to metalloporphyrins, this bi-metallic MOF (CoTPyP-Fe) showed high OER potential [73]. Johnson et al. obtained potential OER catalyst [69].

**Figure 5:**
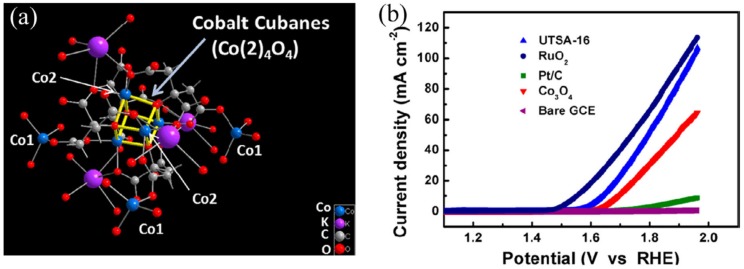
(**a**) Presentation of coordination structure of UTSA-16 (**b**) In 1.0 M KOH solution LSV plots of electrodes modified by UTSA-16, RuO_2_, Co_3_O_4,_ and related materials. Reprinted from Jiang et al. [76] with permission from the American Chemical Society. Copyright (2017), the American Chemical Society.

Lin et al. [70] have also reported OER potential of Ru-UiO-67. Some inorganic catalysts are known for their high OER potential, especially ones incorporating cobalt metal. OER catalysis efficiency of cobalt-based MOF [Co(bim)_2_] (Co-ZIF-9) has been studied [74]. This cobalt MOF is zeolitic frame-work incorporating tetrahedral Co(II) and bidentate ligands. Co-ZIF-9 exhibited low OER potential in potassium phosphate buffer of pH 7 [74]. OER potential of Co-ZIF-9 was also found poor at pH 13. The restricted approach of metal centers may be the reason for the poor OER potential of this cobalt-based MOF [75]. The 3D framework of UTSA-16 consists of Co-ions and tetra-nuclear Co_4_O_4_ units (Figure 5a) [76]. UTSA-16 electrode showed η_10_ = 409 mV and a small TS of 77 mV dec^-1^ (Figure 5b) in 1 M KOH solution, which proved better than standard Co_3_O_4_ counterpart did. The O_2_ evolved during the CPE experiment of UTSA-16 was measured with the help of gas chromatography. MAF-X27-OH showed better OER potential in comparison with MAF-X27-Cl. This enhanced OER potential may be due to the direct involvement of OH^-^ in coordination phenomena [77,78]. By using 0.1 KOH solution, as an electrolyte, thin film-based MOFs were grown on Ni foams following electrochemical reactions [54]. Negligible loss of potential was observed after 15 h of the electrolysis process, and weakness in PXRD intensity suggested some sort of degradation. Co-Fe based polymer has been investigated for the oxidation of water. By using a solution having Ni^2+^, Co^2+^ ions, and benzene dicarboxylic acid (BDC), nanosheets of bimetallic MOF (NiCo-UMOFNs) has been synthesized [79]. For OER potential in 1 M KOH, this MOF (NiCo-UMOFNs) showed low onset potential values and small Tafel slope, which proved superior as compared to Co-UMOFNs, Ni-UMOFNs and other commercial RuO_2_ [80]. In summary, MOFs provide a nice platform to study the structural relationship of OER catalysis. Also, MOFs exhibited excellent OER potential as compared to other catalysts. For improvement of OER potential, further studies are required to optimize the crystal morphology, size, and amount of sample loadings.

## 4. Photocatalysis Perspectives of MOFs

Recently, the development of MOF-based porous composites as photocatalysts has attracted the attention of researchers [34,41,81]. As photocatalysts these materials, possess unique features. For example, the MOFs having metal nodes in their architecture can be transformed into their respective metallic oxide or sulfides during the photocatalytic reactions. Similarly, the reaction between the charge carriers and substrate molecules is greatly influenced by the high porosity of the MOFs-derived materials. These pores facilitate easy access to these active sites, thus significantly quashing the recombination of holes and electrons.

### 4.1. Photocatalytic Breakdown of Dyes or Dyes-Containing Waste Materials

MOF-based composites such as metal oxides or sulfides are known as excellent carriers for the degradation of toxic dyes. The number of publications has reflected this enormous photocatalytic potential of MOFs to degrade or breakdown the dyes or dyes-containing byproducts [82,83]. The hybrid structures of MOF-based oxides and precious metals having a controllable size, shape, and structure are used as an excellent photocatalyst for the degradation of dyes. For example, Fe_2_O_3_/C, Au/ZnO, C–N-doped ZnO, and MOF-based Co_3_O_4_/GN have been proven exceptional photocatalysts for the degradation of the dye in wastewater [84,85,86,87]. Because of its environmentally friendly nature and excellent photosensitivity semiconducting, zinc oxide (ZnO) has been employed for the degradation of organic dyes [88]. So far, many ZnO-based structures have been engineered using ZIF-8 as a building unit. The fabricated catalysts have shown excellent performance in degrading the dyes compared with the traditionally used TiO_2_. Cao and coworkers developed a graphene-based 3-D network for the development of ZIF-8 polyhedrons [89]. The degradation performance of the formed hybrid material was tested against methylene blue (MB). The formed material exhibits excellent degradation ability under UV-radiation in 60 min. This enhanced performance was attributed to the large surface area and effective interaction between the graphene network and the metal oxides. Through the direct carbonization of ZIF-67@ZIF-8 in the presence of an inert atmosphere. Chen and coworkers reported the synthesis of a ZnO@carbon core-shell composite material [90].

It is worth mentioning here that almost all of the as-synthesized metal oxides hybrids carry out the photocatalytic reactions in the presence of UV-light. Because these materials have huge energy gape, therefore the developments of the materials responsive to the visible light are greatly required which can facilely be used as a potential photocatalyst. Recently, Zhu and coworkers have developed MOF-based ZnO composite material incorporated with reduced graphene oxide (RGO) through the microwave-supported technique. The synthesized catalyst has successfully been applied as a catalyst for the degradation of (MB) [91]. Excellent stability and finest photocatalytic activity were revealed by the composite material containing 1.5 wt% RGO as compared with additional composites having different amounts of RGO and pure ZnO. This increased performance was attributed to the improved absorption of visible light and interaction between RGO and ZnO. Moreover, in another approach Pan and coworkers have presented the carbonization of ZIF-8 to develop ZnO–carbon composite material as a photocatalyst [92]. The investigations prove that the carbon contents present inside the material favors the degradation of organic dyes. As the dopant (carbon) efficiently proscribes the recombination of holes and photo-induced electrons. Xu and coworkers carry the carbonization of Co/In–MOF@palygorskite in the air at 500 °C to introduce an In_2_O_3_/Co_3_O_4_ based palygorskite. The attained catalyst displayed superb activity concerning with the organic dye degradation [93].

### 4.2. Photocatalytic H_2_ Production

**Figure 6:**
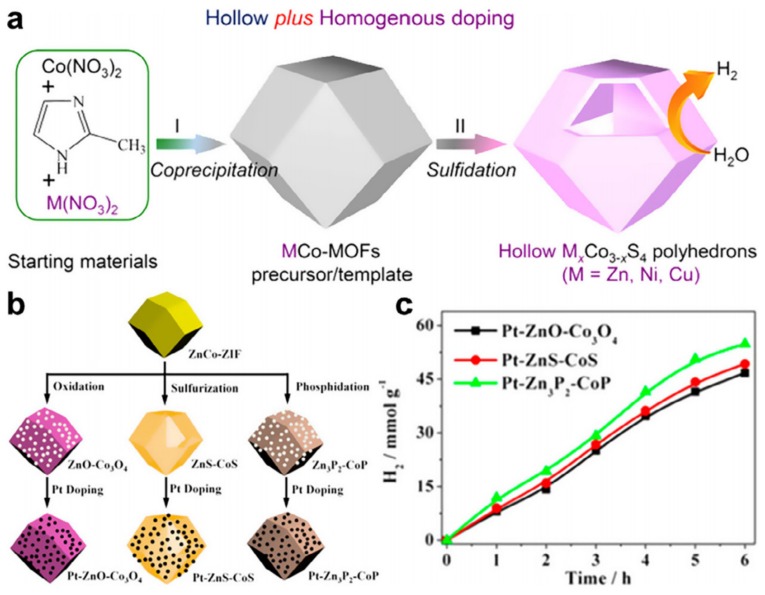
(**a**) Schematic illustration of the preparation of hollow M_x_Co_3-x_S_4_ for photocatalytic hydrogen production. Reprinted from Huang et al. [96] with permission from the American Chemical Society. Copyright (2016) the American Chemical Society (**b**) Schematic illustration of the fabrication of Pt–ZnO–Co_3_O_4_, Pt–ZnS–CoS and Pt–Zn_3_P_2_–CoP photocatalysts (**c**) The comparison of photocatalytic performance of Pt–ZnO–Co_3_O_4_, Pt–ZnS–CoS and Pt–Zn_3_P_2_–CoP. Reprinted from Lan et al. [97] with permission from Elsevier. Copyright (2017) Elsevier Ltd.

The interesting feature of the photocatalyst based splitting of water strongly associated with the bandgap energy, charge transfer/separation properties, types of co-catalysts/active sites, and photo-absorption capability. Recently, several approaches have been extensively demonstrated to enhance charge transfer efficiency and catalytic performance. Titanium dioxide (TiO_2_) is a well-known photocatalyst that has been used traditionally for a long time to carry out various photochemical reactions. However, the TiO_2_ can absorb UV radiation because of the larger bandgap, which is only 5% of the total sunlight. However, TiO_2_ can efficiently catalyze the reduction of water by absorbing UV light. The Fe_2_O_3_ is a better choice in place of TiO_2_ as it can absorb visible light, but the problem is its conduction band is too low for driving the H_2_ generation. Dekrafft et al. presented an effort to solve the problem. The group uses a template-assisted methodology for the purpose. They utilize the nanoscale MOF as a template to fabricate a Ti-assisted nanocomposite structure in which Ti was used as a core-shell. The group carbonizes TiO_2_ coated MIL-101 in the presence of air, which was further used as a support for Pt NPs [94]. The interesting feature of the composite material was the thickness of TiO_2_ that can be controlled by adjusting the reaction time and concentration of the acid. The prepared Fe_2_O_3_@TiO_2_/Pt with the retention of octahedral structure and porosity displayed a linear growth of the production of H_2_ during the complete duration of photocatalytic water reduction. Su and coworkers through the sulfidation of Cd–Fe PBA developed yolk-shell CdS based micro-cubes under microwave-assisted hydrothermal conditions [95]. The exchange of ions between iron complex and sulfide ions during the sulfidation process takes place at the exterior of MOF in the beginning and later on proceeded on the outer layers. In conclusion, the distinctive architecture was obtained, displaying exceptional production of H_2_ photocatalytically. In the same way, Huang and coworkers presented bimetallic MOF for the development of bimetallic sulfides (Figure 6a) [96].

Later, molecular clustar@oxide was synthesized as a catalyst by Lan and coworkers. The group incorporated the pores of ZIF-67 with polyoxometalates (POMs) accompanied by the carbonization in the air [97]. The as-prepared POM@Co_3_O_4_ showed enhanced production of oxygen as compared to the pure MOF-derived nanocomposite. This enhanced performance was because of the improvement in the transfer of electrons from Co_3_O_4_ to POM (Figure 6b) [97]. A hollow polyhedral structure was obtained after successive sulfidation. Further, Xiao and Jiang selected a MOF, MIL-53(Al) as a thermally stable system for the development of the metal sulfide-based system. To construct a replica of metal oxide, various metallic nitrates were incorporated into the hollow structure of MOF. These were converted to respective sulfides during the process of nano-casting inside the MOF [98]. Finally, the hierarchically porous CdS was attained by removing the MOF, and the obtained material displayed a brilliant production of H_2_ photocatalytically. This production rate was far better than the production by using nanosized CdS in bulk as counterparts. This enhanced production owes to the porous and nanosized structure of the material. It is worth mentioning here that the creation of heterojunctions can also increase the photocatalytic activity. Zhang et al. presented an economical and efficient catalyst for the oxidation of water. The group carbonized ZIF-67 to obtain the catalyst, and they named it T–CoO_x_–C [99].

### 4.3. Photocatalytic CO_2_ Conversion

The capability of CO_2_ transformation into green energy is considered an efficient approach [100], which can address the energy crisis. A well-designed porous structure has been constructed by Ye and coworkers. The porous nanocomposite (Fe@C) comprises of ultrathin layers (1–3) of carbon as shell and iron nanoparticles as a core through simple carbonization of Fe–MIL-101 [101]. The energy for the chemical reaction was provided by the nanoparticles as the local temperature of the NPs increases due to photothermal effects, under light irradiation. On the other hand, the selectivity to CO is governed by the compactness of the carbon layers. The production rate of the as-prepared nanocomposite material was 2196.17 mol. [101]. Furthermore, Wang and coworkers prepared the heterostructured hierarchical nanotubes by using In_2_S_3_–CdIn_2_S_4_. The as-prepared material gives excellent performance for the photocatalytic reduction of CO_2_ [102]. The group utilizes the solution infiltration approach accompanied by heat treatment. The transformation of MIL-68 prisms into In_2_S_3_ nanotubes was observed. This, in turn, immediately goes through the process of cation exchange and transformed into heterostructured nanotubes, which support the transformation of CO_2_ photocatalytically.

## 5. Biomedical Applications of MOFs

Chemistry beyond the molecule or supramolecular chemistry has set new standards to design and tailor a range of new molecules [103]. It attracted scientific fascination after the award of the 1987 Nobel Prize for Chemistry to Cram, Lehn, and Pedersen in recognition of their prestigious work in the field. The custom-based design of molecules through supramolecular chemistry has opened new horizons in the field of nanoscience. Nanomaterials such as micelles, iron-based nanoparticles, and liposomes have been reported for target drug delivery, biosensor, and diagnosis and image biosensor. Several arenas where MOFs have been widely used include molecular electronics, luminescence-based sensors, catalysis, gas storage, and separating agents [104]. MOFs have been constructed based on industrial usage, including storage and separation of industrial materials such as CO_2_ and ethyne. Similarly, some other applications include solar energy, liquid-phase separation, proton construction, and air quality control. Some MOFs have been constructed for sensing purposes that are used in optical or colorimetric detection of materials such as aflatoxin, explosive, especially nitro explosive detection, inorganic species, and antibodies. It has also been used for the detection of salicylaldehyde and ribonucleosides [105,106].

Applications of MOFs in biomedicine are quite immature, but this field of research is progressively growing. In mid-2000s, MOF was used for controlled drug delivery and as a contrasting agent in magnetic resonance imaging (MRI). Currently, there are significant interventions in diagnosis and therapeutic in which nanomaterials played a vital role in achieving the desired results. Current studies have revealed that MOFs are emerging areas of interest for researchers serving as a contrasting agent in MRI T1-(spin-lattice relaxation time) or T2-(spin-spin relaxation time) or combined T1- and T2-contrast agents and targeted drug delivery or combinatorial application in both MRI and diagnosis [107]. In various cases, MOFs are modified functionally or structurally by conjugating with other biomolecules as a bio-ligand, biopolymers for specific delivery of drugs. Similarly, some modifications have been carried out to improve MRI imaging, increase stability, and minimize the toxicity [108].

MOFs display a degree of variations that depend upon the chemical nature. MOFs can be designed as (1) pellets or tablets, (2) thin films for patches, (3) composite materials based on polymers or inorganic matrices (silica-covered MOFs, patches, etc.), or stable solutions of nanoparticles with modified surfaces [109]. The image properties of MOFs are generally based on surface modifications with inorganic materials (zirconium, manganese, iron, and organic materials (chitosan, polyethylene-glycol (PEG)) at post-synthetic modification (PSM) phase [110,111].

### 5.1. MOFs for Drug Delivery

In the past decade, MOFs have been among the most promising candidates for targeted drug delivery. As compared to other delivery systems, MOFs have successfully fascinated researchers owing to their versatile properties like high absorptive nature, porous structure, precise drug-release, and storage capability. A wide range of drugs with hydrophilic, hydrophobic, and amphiphilic properties can be easily accommodated in MOFs [18]. Drugs can bind covalently (provide more ability to control the drugs) or non-covalently with MOFs depending on the nature of drugs and the target site. In MOFs, drugs can be tethered through biomolecules or encapsulated in the core of MOFs [112]. For drug delivery, many factors such as pore size, conformation, and pre and post-synthetic modifications can affect the physicochemical properties of MOFs and their drug delivery efficiency. These factors corroborate the capacity of MOFs to carry the drug, absorption properties, stability, and release at the target site. MOFs follow the matrix degradation mechanism instead of other nanomaterials to release the drugs [113]. As discussed above, modification in MOFs exponentially enhances their functional properties, for example, iron-based BioMIL-1 MOFs exhibited up to 75% drug-carrying capacity as compared to traditional MOFs [114]. Previous studies reported various routes of drug delivery encapsulated inside the MOFs including thin film, tablets, some kinds of patches, injections, and pills according to the compliance of the patient [115]. The drug loading methods have been employed through the in-situ method during the synthesis phase (pre and post-synthesis stage). Multimodal and imaging drug delivery system has been attained by the non-covalent bonding of drugs along with covalent bonding on the surface of MOFs. The main advantages of non-covalent bonding are the increased loading capacity of MOFs such as cisplatin and ibuprofen and controlled release at the target site [116]. The use of zeolite and mesoporous silica has minimized the bursting effects, and thus increasing the efficacy of MOFs [117].

**Figure 7:**
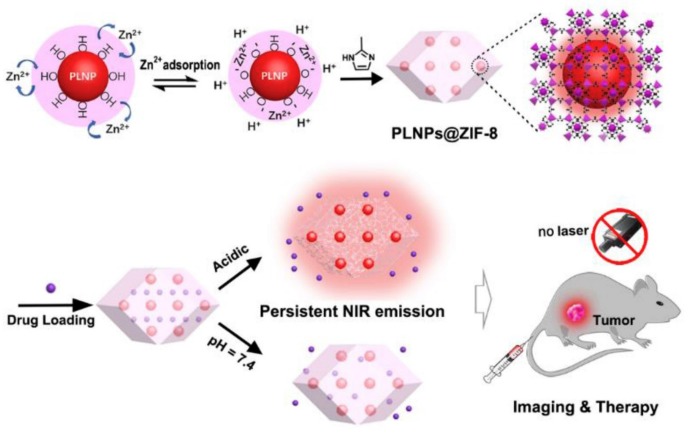
Synthesis of long-lasting NIR persistent luminescent MOF (PLNPs@ZIF-8) for acid-activated Tumor imaging and drug release. Reprinted from Zhao et al. [120] with permission from Elsevier. Copyright (2019) Elsevier Ltd.

Su et al. [118] developed a multifunctional-targeted antitumor DDS based on the nanocomposite of zirconium MOF, UiO-66 embedded with silver nanoclusters using a one-pot encapsulation method. Confocal laser scanning microscopy revealed that aptamer-modified UiO-66@AgNCs@Apt was effectively taken up and internalized by target cancer cells with profound selectivity. In vitro cellular uptake and drug delivery analysis showed that UiO-66@AgNCs@Apt@DOX nanocomposite was internalized by AS1411-driven endocytosis, and the released doxorubicin effectively targeted to the nucleus serving as promising in vivo DDS. Cell compatibility test represents that the synthesized nano-construct exhibited negligible toxicity to MCF-7 cell in a broader concentration range, and the drug preparations exhibit target-oriented delivery of doxorubicin and controlled intracellular release resulting in enhanced and robust in vitro antitumor activity [118]. More recently, a hydrophobic nano DDS was designed using RGD (Arg-Gly-Asp) modified camptothecin@zeolitic imidazolate framework-8 (RGD@CPT@ZIF-8) as a unique MOF for enhanced and targeted cancer therapy [119]. The fabricated nanocarrier has shown the robust cancer cells targeting performance because of the integrated RGD functionality. In addition, the developed nanoplatform exhibited the excellent pH-responsive drug release and generation of ROS for enhanced cancer treatment [119]. Zhao et al. [120] reported a luminescent PLNP@ZIF-8 MOF with durable luminescence of near-infrared, which facilitates tumor site activated luminescence imaging. A surface adsorption induced self-assembly method was used to construct PLNP@ZIF-8 at room temperature (Figure 7) [120]. The PLNP@ZIF-8 MOF produced persistence NIR luminescence for hours or even days and thus enabled background interference-free long-term and deep tissue penetration and imaging without external irradiation. Importantly, the porous framework ZIF-8 shell provided a high anti-cancer drug loading capacity, and acidic tumor site activated drug release was effectively achieved.

### 5.2. MOFs for Sensing and Chemical Catalysis

**Figure 8:**
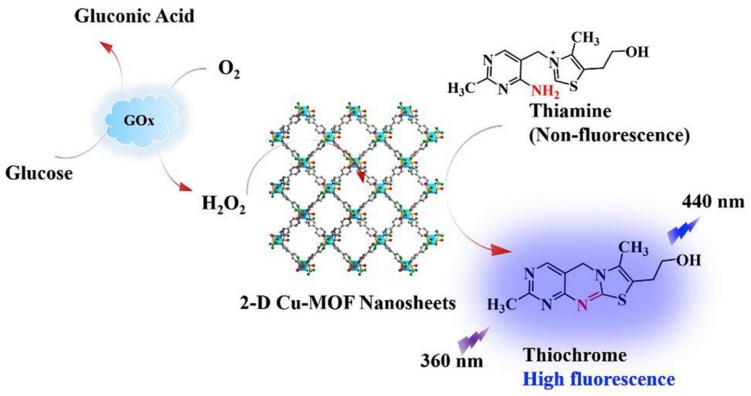
Representation of two-dimensional Cu(bpy)_2_(OTf)_2_ metal-organic framework nanosheets for fluorescent detection for H_2_O_2_ and glucose. Reprinted from Shi et al. [123] with permission from Elsevier. Copyright (2019) Elsevier B.V.

MOFs having luminous properties accompanied by size and shape-selective sorption properties could be regarded as potential bio-sensing devices tools and imaging for disease identification [121]. Although MOFs have been utilized for the detection of various types of gases, the construction of MOFs to sense oxygen, glucose, or other biomolecules occupies a prominent position in biomedical applications. For biosensing applications, magnetism, luminescence, and photo-stability play a dynamic role to define the topographies of MOFs. Additionally, other factors, namely specific coordination, channel size, and degree of chirality are also considered persuasive for biosensing applications [122]. Water-stable two-dimensional MOF based Cu(bpy)_2_(OTf)_2_ nanosheets were used for the fluorescent detection of glucose and H_2_O_2_ with their inherent peroxidase-like catalytic activity (Figure 8) [123]. According to the results, a highly sensitive and selective fluorescent recognition for glucose was achieved under the optimal reaction time, pH, and temperature conditions with a sensing limit of 0.41 μM. In addition, the newly proposed fluorescent method was also exploited for the quantitative measurement of glucose in human serum as a diagnostic indicator for diabetic patients [123].

A highly luminescent europium organic framework, [Eu_2_(MTBC)(OH)_2_(DMF)_3_(H_2_O)_4_] · 2DMF · 7H_2_O designed through solvothermal methods was utilized for the selective and sensitive detection of Cu^2+^ and UO_2_^2+^ among monovalent, divalent, and trivalent metal cations. The detection limits were calculated to be 17.2 and 309.2 μg/L for Cu^2+^ and UO_2_^2+^, respectively. Notably, the detection capacity of the as-synthesized luminescent MOF based probe was much lower than the EPA standard for Cu^2+^ content. Conclusively, this probe can be used to provide insight into creating MOF-based multifunctional sensors for the detection of both Cu^2+^ and UO_2_^2+^ ions [124]. Wang et al. [125] synthesized a series of isostructural lanthanide MOFs (LnMOFs) using solvothermal conditions by fine-tuning the ratio of Eu^3+^ to Tb^3+^ and studied their sensing performances for pH and toxic phenolic compounds. Experimental results demonstrated that these MOFs appeared as promising luminescent sensors for selective detection pH (ranging from 1-6) and *β*-naphthol. Moreover, as-obtained LnMOFs exhibited excellent stabilities and potential photocatalytic performances for the degradation of rhodamine (RhB) under UV light irradiation. Ethanol produced by the sugar cane industry can be used as automotive fuel either in anhydrous form or as hydrated ethyl alcohol fuel (HEAF). The adulteration practice, i.e., the addition of methanol leads to severe complications for consumer’s health and the economy [126]. Therefore, it is extremely important to control the methanol concentration in HEAF. Fonseca et al. [127] developed MOF-76-LnMOF based on an optical sensor for the detection of methanol in HEAF using trimesic acid and terbium as a ligand and metal ion center, respectively. Results revealed that Tb^3+^ luminescence intensity increases with the increasing quantity of methanol in ethanol fuel. Therefore, it might be considered as an applicable sensor to evaluate methanol adulteration in ethanol fuel above the permissible level. Antibiotics are widely applied in numerous applications, and thus can be identified in several places resulting in various ecological menaces. Recently, a three-dimensional MOF {[ZnL(H_2_O)]·2NO_3_}_n_ with excellent stability was constructed based on Zn^II^ and a neutral linker. The as-prepared luminescent sensor possessed marked water stability and excellent quenching performance toward antibiotics in the water system. Moreover, it can serve as a promising heterogeneous catalyst for the Knoevenagel condensation reaction accompanied by a remarkable recycling capability during the catalytic applications [128].

In the modern area of chemical analysis, researchers found new analytical challenges like specificity, detection limit, and improvement in sensitivity due to the emergence of novel materials [129]. For this purpose, MOFs provide unusual features, especially surface modification to meet the new challenges. MOFs application in analytical chemistry attracted the interest of researchers due to their high range of flexibility and accommodation of certain chemical reactions like cross-aldol condensation, Claisen–Schmidt reaction, acetalization of aldehydes, acid-catalyzed selective hydrogenations, acetalization of aldehydes, and ring-opening polymerization of epoxides. There are following MOFs that have been used for analytical applications such as Al-MCM-41, UiO-66(NH_2_), Fe(BTC), Cu(NO_3_)_2_–3H_2_O and Cu_3_(BTC)_2_ [130].

### 5.3. MOFs for Environmental Remediation

Generally, wastewater effluents are loaded with a variety of hazardous organic pollutants such as bisphenol A (BPA), synthetic dyes, antibiotics (i.e., tetracycline hydrochloride (TC) and *p*-nitro-phenol (PNP) that are assimilated into the soil thus can affect the growth of plants and microorganisms. Cellulose aerogels loaded with zeolitic imidazole framework (ZIF) materials, ZIF-9 and ZIF-12 were used as green and environmentally friendly catalysts for the degradation of TC, RB, and PNP. The as-synthesized aerogels showed high catalytic activity leading to the removal of about 90% PNP within 1 h along with remarkable tolerance to pH. Most importantly, the prepared aerogels can be easily separated from the reaction mixture resulting in excellent reusability without having any impact on PNP degradation [131]. Zirconium-mediated MOFs UiO-66 showed activity as environmental catalysts for the removal of toxic organophosphate compound, dimethyl-4-nitrophenyl phosphate (methyl-paraoxon) in an aqueous environment [132]. Tang and Wang, [133] reported the degradation of sulfamethazine (SMT) by a novel Fenton-like catalyst, MIL-100(Fe) with coordinatively unsaturated iron center (CUS-MIL-100(Fe)). Various operating parameters, including H_2_O_2_ concentration, pH, catalyst amount, and initial concentration of SMT were investigated to achieve the optimized catalytic performance. Results demonstrated that as-prepared CUS-MIL-100(Fe) catalyst led to complete degradation of SMT within 3 h under the optimal conditions of pH 4.0, SMT 20 mg L^−1^, H_2_O_2_ 6 mM, and catalyst 0.5 g L^−1^. This elevated catalytic activity might be attributed to the formation of mesopores, large specific surface area, and integration of coordinatively unsaturated metal sites. In addition, CUS-MIL-100(Fe) presented excellent reusability and stability. MIL-96 MOF produced by methanol (MIL-96-Me) as a modulator can be recycled for the elimination of water pollutants with a greater adsorption capacity of *para*-hydroxybenzoic acid (*p*-HBA). The highest p-HBA adsorption was recorded to be 521 mg/g, and a possible adsorption mechanism was advocated depending on pore-filling, solution chemistry of p-HBA, and functional groups of MIL-96 [134]. Additionally, the samples were carbonized to develop robust Al_2_O_3_/C composite, which exhibited complete catalytic degradation of an organic pollutant, methyl orange within 15 min, representation admirable composite activity in catalytic reactions [135]. Sulfachloropyradazine (SCP) is one of the sulfonamide antibiotics which is extensively applied in animal husbandry. The identification of antibiotics in sewage wastewater is of profound concern and has appealed considerable researcher’s attention to mitigate harmful chemicals from wastewater, including SCP and p-HBA. Peroxymonsulfate was successfully activated by an extremely active and water-stable Co-based MOF, bio-MOF-11-Co, and applied for mitigation of water contaminants, including *para*-hydroxybenzoic acid (*p*-HBA) and sulfachloropyradazine (SCP). Both water pollutants were rapidly degraded by the synthesized bio-MOF, which can be recycled several times without dropping the catalytic activity by simply water washing. The reaction kinetics was reinforced in the presence of electron-rich nucleobase adenine by electron donation accompanied by cobalt atoms in the structure of bio-MOF [135]. Ferrocene (Fc) appeared as a heterogeneous catalyst for Oxone activation to produce sulfate radicals (SR) that result in the degradation of organic contaminants. Zhang et al. [13] selected a novel Fe-containing MOFs designated as MIL-101 as carrier support for the chemical immobilization of Fc. Catalytic potentialities of Fc-MIL easily synthesized via the Schiff base reaction for Oxone activation were tested using batch reactions of amaranth dye degradation [13]. Results revealed that Fc-MIL displayed exceptional catalytic activity for the degradation of amaranth dye. In contrast to earlier reported catalysts, Fc-MIL showed a very lower energy of activation, and thus proved its robustness over other catalysts for environmental mitigations.

### 5.4. MOFs for Antimicrobial Applications

Bacterial infections are considered as a worldwide community health concern and pose a substantial economic and health impacts in many developing countries. Gram-positive (*S. aureus*, β-hemolytic *Streptococcus*) and Gram-negative bacteria (such as *P. aeruginosa*, *E. coli*) are responsible for causing most human infections. An anticipatory control of drug resistance bacterial infections is presumably one of the most pertinent approaches to diminish the disease occurrences. In this juncture, MOFs have recently emerged as potent antimicrobial agents that could be ascribed to the incorporation of metallic contents, such as Ag, Ca, Cu, Fe, and Zn [136,137,138]. Zinc MOF (Zn@MOF) was synthesized and investigated as a novel carrier for volatile antimicrobial essential oils by incorporating thymol with a loading rate of 3.96%. The thymol-incorporated Zn@MOF was evaluated to inhibit a mixture of three bacterial strains. The synthesized T-Zn@MOF was found to be highly effective in inhibiting *E. coli* O157:H7 without a logarithmic growth phase in 24 h due to the continuous release of thymol [139]. The antimicrobial activities of three Zn-mediated nano MOFs (nMOFs)–namely IRMOF-3, MOF-5, and Zn-BTC were investigated both individually as well as in combination with kanamycin and ampicillin antibiotics. According to the results, the nMOF/drug formulations revealed substantially enhanced inhibitory activity against four bacterial strains, including *E. coli*, *S. lentus*, *S. aureus*, and *L. monocytogenes* in comparison to the individual nMOFs or antibiotics [140]. The nano MOF system enables antimicrobial activity via regulation or inhibition of enzymes associated with biosynthesis of the cell wall, metabolism, and repair of nucleic acid, protein synthesis, and interference in membrane structural organization. It generally results in physical impairment of bacterial cells or disorganization of the plasma membrane. As compared to other well-known analogous metal/metal oxide nanoparticles, the MOFs can serve as a metal ions reservoir, which is gradually released leading to a continuous antibacterial activity [140]. A new type of cobalt-based MOF containing polylactic acid (PLA) fibers composite was assessed for antimicrobial activity against *Staphylococcus aureus and Pseudomonas putida*. Experimental findings revealed high sensitivity of *S. aureus* to cobalt-based fibers with up to 60% reduction in CFU with regard to pristine PLA mats. The occurrence of viable but non-cultivable microorganisms with no ability to form colonies was also observed [141].

### 5.5. MOFs for Gas Storage and Separation

Storage and separation of gases in medical and agriculture have a large range of applications. Large surface area to pore size plays a role in increasing the capacity of gas storage of MOFs. For example, M-CPO-27-based MOFs have a distinct aptitude and high efficiency in transporting medical gases like hydrogen sulfide and nitric oxide. Similarly, MOFs (HKUST-1) have been reported as highly applicable to the storage and delivery of NO gas [142]. Another emergent application in agriculture is to separate, control and store the greenhouse gases (CO_2_), toxic gases (CO and NH_3_) as well as energy-related gases (H_2_ and CH_4_) that are emitted from greenhouse effect [143]. A new class of MOF (UTSA-220, L = (1*E*,2*E*)-1,2-bis(pyridin-4-yl-methylene)hydrazine) displays dual properties of best pore size along with robust binding sites for acetylene. It shows significantly greater C_2_H_2_ uptake capacity as compared to other light hydrocarbons. Abdolalian and Morsali, [144] synthesized and characterized a breathing MOF followed by the introduction of TMU-42 as one of the highest CO_2_ storage capacity and surface area in pillared-MOFs. TMU-42 displayed a breathing phenomenon in CO_2_ uptake that is attributed to a pressure-dependent pore opening-closing process and flexibility of the framework. It also showed insignificant adsorption of N_2_ due to reversible, selective, and hysteretic CO_2_ adsorption. Huelsenbeck et al. [145] evaluated the adsorption characteristics of anisotropic [Zn_2_(NDC)_2_(DABCO)]_n_ MOF for the separation of CO_2_/CH_4_ gas by controlling the MOF crystals morphology with modulators. Equilibrium data shows a slight selectivity towards CO_2_, while lower diffusion time constants were observed for CO_2_ than CH_4_ using kinetic analysis. Recently, tetra-carboxylic ligand-based porous heterometallic MOFs [In_6_O_3_Tb_3_O(CBDA)_3_]·18DMF·3H_2_O (In/Tb-CBDA) (CBDA = 5,5′-(carbonylbis(azanediyl))-diisophthalic acid, DMF = *N*,*N*-dimethylformamide) of high chemical stability were solvothermal synthesized by adopting a heterometallic cooperative crystallization strategy [146]. The assembled frameworks maintained high stability under the heating conditions of 150 °C, air contact, and exposure to acidic and alkaline solutions for 12 h. In addition, the heterometallic MOFs possessed highly efficient and selective adsorption of C_2_H_6_/CH_4_, CO_2_/CH_4_, and C_3_H_8_/CH_4_ at room temperature according to the calculation of theoretical ideal adsorption solution theory [146]. Chen and coworkers, [147] used an environmentally benign and promising method to fabricate a highly stable aqueous solution-based zirconium MOF, UiO-66-NO2, at room temperature. We evaluated the phase purity, porosity, thermal stability, particle morphology, and size of the resulting material. Enhanced uptake and excellent repeatability of water and ethanol vapor isotherms make UiO-66-NO_2_ as a robust adsorbent in water harvesting or adsorption-based cooling applications.

### 5.6. MOFs for Biomimetic Catalysis

Natural enzymes display many critical drawbacks such as elevated cost, storage and recyclability difficulties, and loss of activity environmental perturbations, including temperature, pH, and inhibitors. Significant effort has recently been made to extend the application of natural enzymes to enzyme mimetics with distinct catalytic performance and high substrate specificity under mild environments. A variety of enzyme mimetics has been designed in the last decade, such as serine proteases mimetics, cytochrome P450 mimetics, etc. Among these, several peroxidase mimetics, hematin, hemin, and porphyrin have received broad-spectrum interest owing to their versatile utilization in detecting glucose and H_2_O_2_ [148]. Qin et al. [149] successfully synthesized a novel composite material by means of an amino-containing MOF to anchor hemin and pretend the peptidic microenvironment of the native peroxidase. The as-prepared biocatalytic system presented peroxidase-like activity by carrying out the oxidation of 3,3,5,5-tetramethylbenzidine (TMB) resulting in a blue-colored solution [149]. A highly selective and sensitive method was obtained for the detection of glucose using this newly developed mimetic peroxidase catalyst with a linear glucose detection range from 1.0 × 10^−5^ mol L^−1^ to 3.0 × 10^−4^ mol L^−1^.

**Figure 9:**
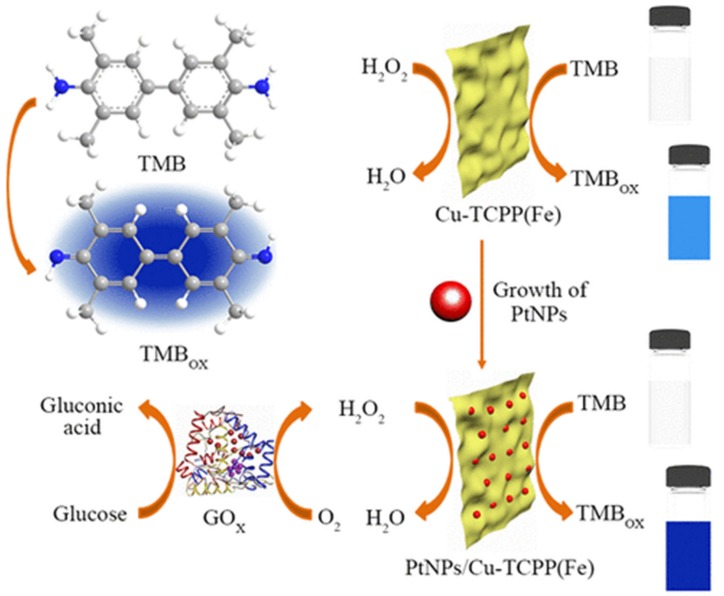
Schematic illustration of the synthesis method of PtNPs/Cu-TCPP(Fe) hybrid nanosheets and its application in colorimetric detection of H_2_O_2_ and glucose. Reprinted from Chen et al. [2] with permission from the American Chemical Society. Copyright (2018) the American Chemical Society.

A simple and promising surfactant-assisted method was developed to fabricate platinum nanoparticle (PtNP)-decorated 2D-MOF nanosheets using a ligand with heme-like structure, Fe(III) tetra(4-carboxyphenyl)porphine chloride. The prepared hybrid nanocomposites showed excellent peroxidase-mimetic catalytic activity owing to their unique structure in contrast to PtNPs, and Cu-TCPP(Fe) nanosheets (Figure 9) [2]. Similar to natural HRP, the catalytic potentiality of hybrid MOF nanomaterials was dependent on temperature, pH, and concentration of hydrogen peroxide. As compared to native HRP, the synthesized MOF-based nanosheets displayed a greater catalytic ability to TMB accompanied by the advantages of low-cost, facile preparation, and catalytic stability. Moreover, a colorimetric method was developed for rapid and sensitive recognition of H_2_O_2_ owing to the excellent peroxidase-mimetic ability of the nanosheets. By integrating with GOx enzyme, a cascade colorimetric method was also proposed for glucose detection with noteworthy selectivity and sensitivity [2]. Yu and coworkers, [150] fabricated an electrode by combining a MOF (PCN-222(Fe)) and acetylene black and applied for sensitive and selective detection of tetrabromobisphenol-A (TBBPA). The prepared PCN-222(Fe) presented exceptional peroxidase-mimetic activity, and resultantly enhanced the electrocatalytic performance for TBBPA oxidation. It intensively adsorbed TBBPA from aqueous media and thereby improved the sensitivity of the sensor because of its large pore volume and huge surface area. Due to its extremely low detection limit, the developed sensor was effectively used in real water samples as a promising alternative for the detection of TBBPA.

## 6. Conclusions, Challenges, and Directions

Despite the remarkable progress seen in recent years for catalytic applications of MOFs and MOFs-derived composites/materials, many challenges still exist that need to be solved: (i) It is still far from possible to design and build inexpensive MOFs at large-scale with high yields, although some “star” MOFs, such as ZIF-8, MIL-100, HKUST-1, etc., have been commercialized. New organic linker’s needs to be created by organic synthesis, and some metal precursors are very expensive. Overall, the development of MOFs is generally a cumbersome and energy-intensive process; (ii) The inherent self-assembly mechanism of MOFs is still uncertain. Therefore, a clear understanding of the growth mechanism(s) is highly essential to construct MOFs with a targeted framework, amendable composition, and uniform or hierarchical pores; (iii) The mechanical, thermal, and chemical stability of MOFs is not adequate. This is the most critical aspect of the catalysts for maintaining catalytic performance and selectivity under real-time catalytic reaction environments. Similarly, the mechanical steadiness of catalysts is indispensable for practical catalysis; (iv) Mass transfer limitations might occur for larger products and substrates because of the microporous nature of the MOFs or MOFs-based materials. The construction of hierarchically porous MOFs could be a unique solution to address this issue. (v) The MOF-assisted catalytic derivatives necessitate the supplementation of some sacrificial mediators to promote imperative redox-mediated photocatalytic reactions, such as oxygen evolution, CO_2_ reduction, H_2_ production, etc. Significant investigations should be carried out in this direction to overcome this issue; (vi) Enhanced conductivity of MOFs is highly desired for rapid transfer of charge in electrocatalytic and photocatalytic applications; (vii) Innovative and state-of-the-art characterization approaches should be implemented to comprehend better the (bio)catalytic transformation processes and other associated mechanisms; (viii) Though a substantial advancement has been made in designing and fabricating MOF-based nanostructured materials, the inherent conversion mechanism remains unclear, which severely hamper the rational fabrication of MOF-based nanocomposites for purposeful applications.

Based on the extensive literature survey, it can be summarized that the synthesis of MOFs and MOF-derived materials/composites for diverse catalytic applications has entered a new phase, where opportunities and the challenges coexist. With the continuous research investigations to overcome the above-described challenges, we believe and look forward to realizing industrial-scale production and real-time exploitation of MOFs and MOF-based novel nanomaterials. Supreme inheriting attributes including large porosity, exceptional surface area, the occurrence of plentiful active species, tunable flexibility and adjustable porous structure make MOF-based porous constructs with a bright future for (bio)catalysis, energy conversion and storage, environmental remediation, biomedical applications, as well as, the manufacturing of added value bioproducts.
